# Magnitude of vancomycin-resistant *Enterococci* colonization and associated factors among people living with HIV

**DOI:** 10.1093/jacamr/dlaf099

**Published:** 2025-06-11

**Authors:** Abebe Dawud, Tadesse Shume, Rajesh Sarkar, Mandie Maru, Olifan Getachew Wakjira, Ajay Kumar Prajapati, Dadi Marami, Tewodros Tesfa

**Affiliations:** Department of Medical Microbiology, Hiwot Fana Comprehensive Specialized University Hospital, Haramaya University, Harar, Ethiopia; Department of Medical Microbiology and Immunology, College of Health and Medical Sciences, Haramaya University, Harar, Ethiopia; Department of Medical Microbiology and Immunology, College of Health and Medical Sciences, Haramaya University, Harar, Ethiopia; Department of Medical Microbiology, Hiwot Fana Comprehensive Specialized University Hospital, Haramaya University, Harar, Ethiopia; Hiwot Fana Comprehensive Specialized University Hospital, School of Medicine, College of Health and Medical Sciences, Haramaya University, Harar, Ethiopia; Department of Laboratory Medicine, Central Hospital, South Western Railway, Hubli, Karnataka, India; Department of Medical Microbiology and Immunology, College of Health and Medical Sciences, Haramaya University, Harar, Ethiopia; Department of Medical Microbiology and Immunology, College of Health and Medical Sciences, Haramaya University, Harar, Ethiopia

## Abstract

**Background:**

The limited alternatives for managing vancomycin-resistant Enterococci (VRE) have caused concern in the global community for severe infectious diseases. Therefore, the purpose of the study was to determine the magnitude of VRE colonization and associated factors among people living with HIV at Hiwot Fana Comprehensives Specialized University Hospital, Eastern Ethiopia.

**Method:**

A cross-sectional study was conducted among 260 patients on anti-retroviral therapy (ART) who attended the ART clinic from 10 April to 10 May 2022. A structured questionnaire was used to gather information. The stool sample was collected and processed by standard microbiological techniques. The Bile Esculin Azide Agar and blood agar were used to isolate *Enterococci* species (spp.). The antimicrobial susceptibility testing of the isolates was performed using the Kirby–Bauer disk diffusion technique on the Mueller–Hinton agar plate. Data were analysed using Statistical Package for the Social Sciences 25 software. The chi-square (*χ*^2^) test was used to determine the strength of the association. A *P*-value of  ≤ 0.05 was considered statistically significant.

**Result:**

Out of the 260 participants, colonization of *Enterococcus* spp. from stool specimens were observed among 68 (26.2%). Of these, five were VRE. Multidrug resistance was noted in 60 (88.2%) of the *Enterococcus* spp. isolates. Previous antibiotic treatment was significantly associated with VRE (*χ*^2^ = 7.063, *P*-value = 0.009).

**Conclusion:**

VRE must be regularly monitored for antimicrobial susceptibility testing and surveillance to prevent the spread of antibiotic resistance.

## Introduction

*Enterococci* group particularly *Enterococcus faecium* and *Enterococcus faecalis* are the most common causes of urinary tract infection, inflammation of the lining of the heart and its valves, intra-abdominal abscesses, wound infections, bacteraemia and sepsis in humans.^1^  *Enterococcus* species (spp.) are recognized as predominant nosocomial pathogens. It is also a leading opportunistic hospital pathogen.^2,3^

*Enterococcus* spp. are incredibly antibiotic-resistant and pose a serious risk to public health, as one of the main causes of nosocomial infections.^4^ The emergence of vancomycin-resistant Enterococci (VRE) has alarmed the global infectious diseases community as few options are left for disease management.^5^ The *Enterococcus* spp. as a natural commensal and colonized normally makes it difficult to treat VRE infections.^6^ The *Enterococcus* spp. may acquire resistance through *van*-associated genetic elements (*vanA, vanB, vanD, vanE, vanG and vanL*); of which *vanA* and *vanB* are the most prevalent genotypes in clinical isolates. The *vanA* and *vanB* gene clusters are most commonly found in *E. faecium* and are increasingly reported throughout the world. Other transposable elements are also reported to be involved in the spread of antimicrobial resistance.^7^ The risk factors for VRE infection or colonization are often associated with people living with HIV (PLWH), diabetes, renal failure, gastrointestinal disorders, previous antibiotics exposure, age, hospitalizations, or healthcare providers and others.^8,9^

Individuals with weakened immune systems, such as PLWH, are especially vulnerable to VRE infections. This is because this population group is frequently admitted to medical facilities, increasing their risk of contracting VRE.^2,10,11^ There is not enough information about VRE among the study population in the study area. Therefore, this study was designed to determine the prevalence of VRE, associated factors and antibiotic susceptibility patterns among PLWH attending anti-retroviral therapy (ART) clinics at Hiwot Fana Comprehensives Specialized University Hospital (HFCSUH), Eastern Ethiopia.

## Materials and methods

### Study setting and area

A hospital-based cross-sectional study was conducted at HFCSUH from April 2022 to June 2022. The ART clinic at HFCSUH has been providing healthcare services to more than 60 patients daily.^12^

### Study populations

Our study populations include all PLWH patients visiting Hiwot Fana Specialized University Hospital attending the ART clinic. Any human immunodeficiency virus (HIV)-infected patients attending the ART clinic at Hiwot Fana Specialized University Hospital at the time of data collection were included in the study. However, critically ill patients and patients who were taking antimicrobial treatment within the last 2 weeks were excluded from the study.

### Sample size determination and sampling techniques

A single population proportion formula was used to calculate a sample size by taking the prevalence of VRE colonization among PLWH from a study conducted in Dessie Hospital, Ethiopia (5.9%),^13^ with a margin of 0.03 and a *Z* score for 95% confidence interval of 1.96, and finally, a 10% non-response rate was added. The final sample size was 260. A study participant was selected by using a simple random sampling technique. Sampling was conducted using the logbook of ART patients. Participants were selected using a random number table, based on entries in the selection logbook.

### Data collection

A structured questionnaire was developed from different literature works to collect data associated with VRE colonization.^14–16^ The questionnaire contained two parts: socio-demographic variables (age, sex, marital status, educational level, and residence) and associated risk factors [antibiotic treatment history, CD4 count, and Highly Active Anti-Retroviral Therapy (HAART) duration (years), white blood cell count (WBC), alcohol consumption, ART stage, and platelet count].

### Specimen collection

Patients were instructed and provided wide-mouthed, sterile plastic containers to bring about 5–10 g of stool specimens. The collected stool was transported to the Microbiology Laboratory of Haramaya University using the Carry Blair Transport Medium (Oxoid Ltd, UK). Immediately after sample collection, approximately 1 g of the faecal sample was placed on a medium and stored at 2°C–8°C, until transport to the laboratory for processing.

### Bacterial culture and identification

Stool samples were streaked on Bile Esculin Azide Agar (BEA) (Hardy Diagnostics, Santa Maria, USA), and blood agar was incubated for 24 h at 37°C. Plates were observed for the appearance of characteristic colonies with dark halo center and grey colonies. Characteristic colonies were randomly chosen to identify *Enterococcus* spp. through Gram stains, catalase test, salt tolerance test and heat tolerance test.^17^ Only those plates showing Gram-positive cocci in pairs or short chains were subjected to further examination. In addition, the catalase test was carried out on suspected colonies, with only microbial growth that tested negative for catalase production being considered for further analysis. Similar colonies from each plate were chosen, inoculated into Brain Heart Infusion (BHI) broth containing 6.5% salt (NaCl) as they are halophilic, and then placed in an incubator at 37°C for 24–48 h. Growth in the medium was indicated by turbidity. Furthermore, the picked colonies were also inoculated into BHI broth and incubated at 45°C for 24 h to check for growth, which was confirmed by turbidity in the medium. An isolate that met the criteria mentioned above was classified as an *Enterococcus spp.* To facilitate additional identification, the subculture was introduced into BHI broth with 15% glycerol and stored for a short time at −20°C.^17^

### Antimicrobial susceptibility tests

The antimicrobial susceptibility testing of *Enterococcus* spp. Isolates were performed by using the Kirby–Bauer disk diffusion technique as described by the Clinical and Laboratory Standards Institute (CLSI).^18^ From the growth in the agar medium, three to four selected colonies were transferred to a tube containing 5 mL of normal saline (0.85%) and mixed gently to make a homogenous suspension. The turbidity of the suspension was adjusted to a McFarland 0.5 standard. A sterile cotton swab was used to streak the plates. The excess suspension was removed by gentle pressing and rotation of the swab against the tubes inside the wall surface. The swab was used to distribute the bacteria evenly over the entire surface of the Mueller–Hinton agar (MHA). The inoculated plates were kept at room temperature to dry for 3–5 min. With sterile forceps, the following concentration of antibiotic discs was put onto the surface of MHA; the plates were incubated at 37°C for 24 h. Antimicrobial discs such as Penicillin (10 IU), Ampicillin (10 µg), Tetracycline (30 µg), Doxycycline (30 µg), Ciprofloxacin (5 µg), Vancomycin (30 µg), Erythromycin (15 µg), Gentamicin (30 µg) and Chloramphenicol (30 µg) were used to determine antimicrobial susceptibility patterns. The results were interpreted according to the interpretive inhibition zone described by CLSI. Antibiotics are selected based on CLSI recommendation, local availability (in a health facility) and feasibility (cost and method of antimicrobial susceptibility test).^18^ In this study, the dependent variable was VRE colonization. The independent variables were socio-demographic characteristics, behavioural factors and clinical factors.

### Data quality assurance

Data quality was maintained at different stages of the activities of the study. One week before the actual data collection, a pre-test was conducted on 5% HIV-infected patients in the ART clinic of Jugol General Hospital. Culture media were prepared by following the manufacturer’s instructions and the sterility of the media was checked by incubating 3–5% of the batch at 37°C overnight and observing for growth. Pre-analytical, analytical and post-analytical stages of quality assurance and Standard Operating Procedures (SOPs) were strictly followed as per CLSI guidelines. The reference bacterial strains such as *Staphylococcus aureus* American Type culture collection (ATCC) 25923 and *E. faecalis* ATCC 51299 were obtained from Hararghe Health Research Laboratory Ethiopia and used as a control in each batch of microbial culture. MHA with antimicrobial discs was used to ensure the testing performance of the media and antimicrobial discs. To normalize the density of inoculums of bacterial suspension for susceptibility testing, 0.5 McFarland standards were used. As far as the researchers are aware, there isn't enough information about VRE in the study population in the study area. To ascertain the prevalence of VRE among HIV-positive patients as well as their pattern of antibiotic susceptibility, the study set out to identify the factors that are linked to VRE infections among HIV-positive patients in ART clinics located in HFCSUH.

### Statistical analysis

Data were entered into Epi data version 4.6.0.2 software, and analysed by Statistical Package for Social Science (SPSS) program version 26 (IBM, USA). Frequencies and cross-tabulations were used to summarize data. Categorical variables were summarized by using frequency distribution tables. Chi-square (*χ*^2^) was used for categorical variables and Fisher’s exact two-tailed tests were used when the expected value was less than 5 to determine the strength of association between dependent and independent variables. A *P*-value of  ≤ 0.05 was considered statistically significant.

### Ethical considerations

Ethical approval was obtained from Haramaya University, College of Health and Medical Sciences Ethical Review Board with reference number IHRERC/044/2021. Informed consent was taken from patients. Confidentiality of any information related to the patient and their clinical history was maintained. Those PLWH who were positive for *Enterococcus* spp. were reported to clinicians for treatment purposes.

## Results

### Socio-demographic and clinical characteristics of the participants

A total of 260 study participants were enrolled. The mean age and standard deviation were 39.28 ± 10.85 years, and the age range was from 12 to 72 years. About 200 (76.9%) participants were females. The majority of the study participants (91.5%) were urban residents. Around one-third (88/260, 33.8%) of the study participants had attained at least secondary education. About 107/260 (41.2%) of the study participants were unmarried. Regarding clinical characteristics, two-thirds (181/260, 69.6%) of the participants had taken antibiotic treatment previously for more than 2 weeks. A total of 187/260 (71.9%) participants have not been hospitalized before. Most (208/260, 80%) participants had a CD4 count above 350 and almost all participants (258/260, 99.2%) were using ART. Moreover, the majority of the study participants (*n* = 246, 94.6%) had ART duration of >3 years. The majority of the study participants (*n* = 200, 76.9%) had normal haemoglobin levels and most of the participants 90.8% were in HIV Stage 1 (Table 1).

**Table 1.** Socio-demographic and clinical characteristics of the participants colonization among HIV-infected patients attending ART Clinic at HFCSUH, Eastern Ethiopia

Variable	Categories	Frequency	Percentage, %
Sex	Male	60	23.10
	Female	200	76.90
Age	≤20	23	8.80
	21–30	25	9.60
	31–40	98	37.70
	41–50	88	33.80
	≥50	26	10.10
Educational status	Can’t read and write	55	21.20
	Primary (Grades 1–4)	38	14.60
	Junior (Grades 5–8)	79	30.40
	Secondary (Grades 9–12)	77	29.60
	College or University	11	4.20
Resident	Urban	238	91.50
	Rural	22	8.50
Marital status	Single	107	41.20
	Married	100	38.50
	Divorced	41	15.80
	Widowed	12	4.60
Previous antibiotic treatment (>2 weeks)	Yes	181	69.60
	No	79	30.40
History of hospitalization	Yes	73	28.10
	No	187	71.90
CD4 count	≤350	52	20
	>350	208	80
ART using	Yes	258	99.20
	No	2	0.80
ART duration	≤3 years	14	5.40
	>3 years	246	94.60
WBC count	Normal	176	67.70
	Low	61	23.50
	High	23	8.80
Haemoglobin level	Normal	50	19.10
	Low	200	76.90
	High	10	3.80
WHO HIV Stage	I	236	90.80
	II	18	6.90
	III	4	1.50
	IV	2	0.80
Alcohol drinking	Yes	13	5
	No	247	95

### Vancomycin-resistant *Enterococcus* spp. colonization rate

From the total of 260 study participants, 68 (26.2%) of patients showed an *Enterococcus* spp. colonization. Among these isolates, 5 (7.4%) were VRE. Among the isolated *Enterococcus* spp., the majority (62/68, 91.2%) were confirmed resistant to Penicillin. Moreover, a larger proportion (56/68, 83.8%) of *Enterococcus* spp. isolates were reportedly resistant to Ampicillin and Tetracycline. Moreover, the VRE exhibited 100% (5/5) resistance to Ampicillin and Tetracycline.

### VRE-resistant patterns and MDR

**Figure 1.**
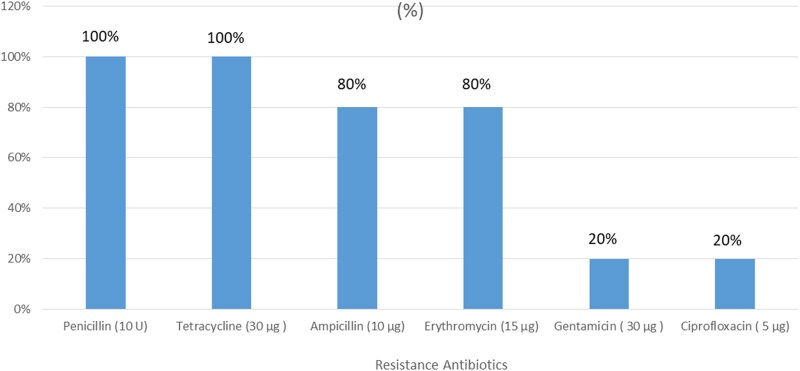
VRE-resistant patterns among HIV-infected patients attending ART clinic at Hiwot Fana Comprehensives Specialized University Hospital, Harar, Eastern Ethiopia. VAN, vancomycin; P, penicillin; TTC, tetracycline; ERY, erythromycin; CYP, ciprofloxacin; R, resistance and R0, sensitive to all; R1, resistance to one; R2, resistance to two; R3, resistance to three.

**Figure 2.**
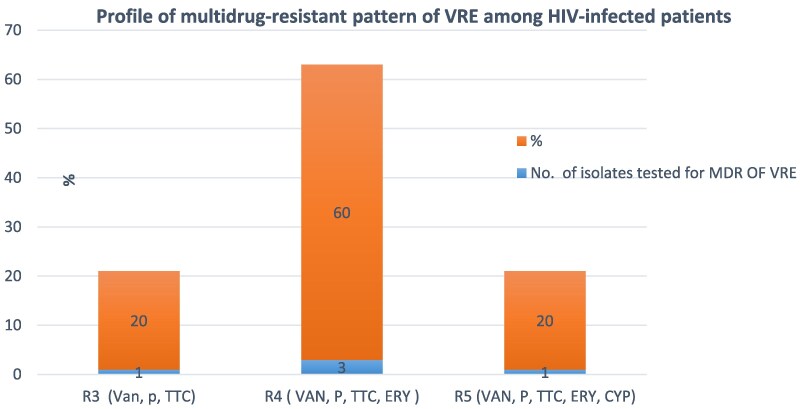
Profile of multidrug-resistant pattern of VRE among HIV-infected patients attending ART clinic at Hiwot Fana Comprehensives Specialized University Hospital, Harar, Eastern Ethiopia.

All five VRE isolates exhibited resistance to Penicillin and Tetracycline, as shown in Figure 1. There was an instance of 88.2% of multidrug-resistant VRE. Likewise, three isolates exhibited resistance to four modern antibiotics, including Vancomycin, Penicillin, Tetracycline and Erythromycin (R4, 60%). Moreover, there is one isolate that shows resistance to five different types of antibiotics: Vancomycin, Penicillin, Tetracycline, Erythromycin and Ciprofloxacin, accounting for 20% of the cases (R5) (Figure 2).

### Associated risk factors for the colonization of VR

Multiple risk factors related to the prevalence of VRE were evaluated and found to be insignificant. These factors included socio-demographic characteristics such as age, gender, educational level, residence and marital status, as well as behavioural and clinical factors like hospital admission, CD4 count, platelet count, WBC count, WHO staging of HIV, and haemoglobin level. The other non-significant risk factors were: history of antibiotic usage, duration of HAART, and alcohol intake. However, previous antibiotic pre-treatment was significantly associated with the VRE colonization rate among ART patients (*P*-value <0.001). However, the details of the antibiotic regimens during pre-treatment were unknown to the researchers. MDR is defined in this study as resistant to ≥1 agent in ≥3 antimicrobial categories (Table 2).

**Table 2.** Chi-square analysis of factors affecting VRE among HIV-infected patients attending ART Clinic at HFCSUH, Eastern Ethiopia

Variable		VRE status				
		Yes	No	χ^2^	df	*P*-value
Sex	Male	1(5.6)	17(94.4)	0.597	1	0.503
	Female	4(8)	46(92)			
Educational status	Illiterate	1(7.1)	13(92.9)	2.289	4	0.732
	Primary 1–4	0(0)	8(100)			
	Junior 5–8	2(7.4)	25(92.6)			
	Secondary 9–12	2(9.1)	20(90.9)			
	College	0(0)	2(100)			
Resident	Rural	5(8.5)	54(91.5)	2.709	1	0.127
	Urban	0(0)	9(100)			
Marital status	Single	1(3.3)	29(96.7)	0.581	3	0.917
	Married	2(7.7)	24(92.3)			
	Divorce	2(22.2)	7(77.8)			
	Widowed	0(0)	3(100)			
Previous Antibiotic treatment (>2 weeks)	Yes	5(8.9)	51(91.1)	7.063	1	0.009
	No	0(0)	12(100)			
History of hospitalization	Yes	3(12.5)	21(87.5)	2.375	1	0.157
	No	2(4.5)	42(95.5)			
CD4 count	≤350	1(5.6)	17(94.4)	2.41	1	0.157
	>350	4(8)	46(92)			
ART duration	≤3 years	0(0)	6(100)	2.138	1	0.207
	>3 years	5(8.1)	57(91.9)			
WBC count	Normal	3(7)	40(93)	3.922	2	0.162
	Low	1(6.7)	14(93.3)			
	High	1(10)	9(90)			
Habit of Alcohol drinking	Yes	3(75)	1(25)	0.151	1	0.748
	No	2(3.1)	62(96.9)			

## Discussion

*Enterococcus* spp. are highly resistant to antibiotics and are a major contributor to infections acquired in healthcare settings, being categorized as a significant public health concern.^4^  *Enterococcus* spp. are known to cause various infections in patients who are immunocompromised or elderly and have serious underlying health conditions.^19^ The rapid rise and growing prevalence of VRE colonization have presented significant challenges for healthcare professionals and authorities, raising serious concerns.^10^

In this study, 26.2% (95% CI: 22.2–31.6%) of individuals were colonized by *Enterococcus* spp., a finding which was in line with the earlier findings from Khartoum, Sudan (22.6%)^20^ and Jimma, Ethiopia (23%),^21^ it was noted that rates were lower in Uganda (17.6%) in stool samples^22^ and notably reduced in Addis Ababa, Ethiopia (3.5%).^23^ The increased occurrence observed in the current study could be attributed to frequent exposure to various antibiotics and immune suppression, known as crucial risk factors for the colonization of *Enterococcus* spp.

In the present study, the prevalence of VRE was recorded at 7.4%, which is in line with the study conducted in North India which reported a prevalence of 7.9%.^24^ In contrast, the result of this study was significantly higher, with reports from Nigeria standing at 42.9%.^25^ Conversely, a similar prevalence was documented in West Amhara, Ethiopia, at 7.7%,^10^ as well as in Gondar, North Ethiopia, at 7.8%^14^ and Dessie Hospital in Ethiopia, at 6.3%.^13^

Moreover, the prevalence of VRE (7.4%) observed in this study was found to be less than that reported in Germany (26.1%),^26^ Brazil (23.4%)^13^ and India (18.6%).^27^ The lower prevalence observed in the current study could be attributed to the diversity in the study population. In contrast, this rate is higher than the 4.7% reported in Chicago, USA.^11^ The discrepancy may be attributed to differences in sample size and the duration of study periods in prior research. However, previous research predominantly focused on hospitalized patients and critically ill individuals exposed to various antibiotics.^28–31^

In this study, vancomycin-resistant *Enterococcus* spp. had higher resistance to Ampicillin (83.3%). The findings aligned with studies conducted in Gondar, North West Ethiopia (81.6%).^14^ Nevertheless, the percentage is greater compared with the rates documented at Dessie Referral Hospital in Ethiopia (34.8%)^13^ and West Amhara in Ethiopia (20.9%).^10^ This variation could be attributed to the common practice of prescribing broad-spectrum antibiotics to HIV patients.

As per our findings, a large fraction (82.3%) of VRE showed a resistance rate to Tetracycline. This finding is in line with findings from India (84.5%)^8^ and Jimma, Ethiopia (77.3%).^16^ Nevertheless, the rate is elevated compared to the data recorded in Arba Minch, General Hospital Southern, Ethiopia (59.3%)^9^ and West Amhara, Ethiopia (28.6%).^10^

Furthermore, in this study, a high level (88.2%) of multidrug resistance (MDR) was detected among *Enterococcus* isolates. This observation is similar to the research conducted in Iraq (85.7%)^32^ and in Jimma, Ethiopia (89.5%).^5^ Conversely, it was noted that an intermediate level of MDR in studies in Dessie Referral Hospital (29.5%), Ethiopia^13^ and Arba Minch General Hospital in Southern Ethiopia (49.6%).^9^ The variation could be due to the method of drug susceptibility testing, as well as the frequency of antibiotic prescription, which could account for the disparity in these results.^29,33^

Regarding associated factors, the current study revealed that previous antibiotic treatment was significantly associated with VRE (*χ*^2^: 7.093, *P*-value = 0.009). This result is consistent with studies reported in Brazil,^13^ Egypt^8^ and West Amhara, Ethiopia.^10^ This might be due to heavy exposure to antibiotics for a prolonged hospital duration, which may cause the acquisition of resistance genes.^34–38^

### Strengths and limitations

The findings may have significant implications for the rationale of conducting a longitudinal study on the surveillance of VRE and MDR pathogens in similar clinical settings. However, the isolated enterococci were not identified at the species level and no molecular characterization was done due to the limitation of resources. This study has not evaluated some variables such as prior specific antimicrobial treatments and other comorbidities.

### Conclusion

The colonization rate of VRE among HIV patients in HFCSUH was around 7%. However, a large proportion of the isolates (88%) were MDR. Additionally, it was noted that prior treatment with antibiotics showed a significant effect on the VRE colonization rate.

## Data Availability

The datasets generated during and/or analysed during the current study are available upon reasonable request.
